# A novel *EMD* mutation in a Chinese family with initial diagnosis of conduction cardiomyopathy

**DOI:** 10.1002/brb3.1167

**Published:** 2018-12-03

**Authors:** Junfeng Zhou, Hui Li, Xiangping Li, Yonggui Li, Mei Yang, Gaoxing Shi, Danyan Xu, Xiaoliu Shi

**Affiliations:** ^1^ Department of Medical Genetics The Second Xiangya Hospital, Central South University Changsha China; ^2^ Department of Gastroenterology The Second Xiangya Hospital, Central South University Changsha China; ^3^ Department of Cardiovascular Medicine The Second Xiangya Hospital, Central South University Changsha China

**Keywords:** conduction cardiomyopathy, *EMD* gene, novel mutation, predominant cardiac phenotype, X‐linked Emery–Dreifuss muscular dystrophy

## Abstract

**Introduction:**

Emery–Dreifuss muscular dystrophy (EDMD) is a hereditary myopathy characterized as triad of muscular dystrophy, joint contractures, and conduction cardiomyopathy. In this study, we diagnosed a X‐linked recessive EDMD patient with severe conduction cardiomyopathy while noteless muscular and joint disorders.

**Methods:**

A Chinese cardiomyopathy family spanning four generations was enrolled in the study. Targeted next‐generation sequencing (NGS) was performed to identify the underlying mutation in the proband and validated by Sanger sequencing. Segregation analysis was applied to all 13 participants.

**Results:**

A novel frameshift mutation (c.253_254insT, p.Y85Lfs*8) of emerin gene (*EMD*) was found and co‐segregated with family members. Other than the typical manifestations of X‐linked EDMD, this patient presented inconspicuous muscular disorders which were later diagnosed after the mutation been identified.

**Conclusions:**

This study enriches the *EMD* gene mutation database and reminds us of the possibility of EDMD while encountering patients with severe heart rhythm defects or dilated cardiomyopathy of unknown etiology, even if they have neither obvious skeletal muscle disorder nor joint involvement.

## 1 INTRODUCTION

Emery–Dreifuss muscular dystrophy (EDMD)—firstly described by Emery and Dreifuss in 1966 (Emery & Dreifuss, 1966)—is a genetic disorder characterized as triad of slowly progressive muscular weakness, joint contractures, and cardiac involvement (Rowland et al., 1979). However, clinical variabilities exit in both interfamilial and intrafamilial patients. EDMD could be inherited as X‐linked recessive, autosomal‐dominant and autosomal‐recessive patterns. The prevalence of X‐linked EDMD is estimated at 1:100,000 (Norwood et al., 2009). The diagnosis of X‐linked EDMD is established in typical triad symptoms and molecular testing with identification of a hemizygous or heterozygous pathogenic variant in *emerin* gene (*EMD*). We described a novel *EMD* mutation in a Chinese family with initial diagnosis of conduction cardiomyopathy.

## 2 MATERIALS AND METHODS

### 2.1 Patient information

We enrolled a cardiomyopathy family spanning four generations from the Department of Cardiovascular Medicine in the Second Xiangya Hospital, Central South University, in Hunan, China. This study was approved by the review board of the Second Xiangya Hospital of Central South University. Written informed consent was obtained from the patient and family members who participated in the study. Peripheral blood was collected from the affected proband and 13 family members.

### 2.2 Mutation analysis

Genomic DNA was extracted from whole blood using a QIAamp Blood DNA Mini kit (Qiagen GmbH, Hilden, Germany). And the exome capture, high‐throughput sequencing, and common filtering were performed in the Sinopath Institute (Beijing, China). All the exomes were sequenced by Illumina HiSeq X Ten platform. About 97.990% of target bases were covered to a total depth of >20× with high‐quality (Q20) reads. The reads were aligned with the human genome reference sequence [University of California Santa Cruz, human genome assembly 19 (UCSC hg19)]. The PolyPhen‐2, SIFT, and Mutation Taster programs were used to predict the effects of mutations on the function of the protein. The conservation analysis was performed by comparing different species amino acid sequences. The pathogenicity of variant was interpreted according to American College of Medical Genetics and Genomics guideline (Richards et al., 2015).

The potential mutations from NGS were validated by Sanger sequencing. Segregation analysis was applied to all participants. Primer pairs were designed by Primer 3, PCR amplifications were performed with forward primer [5’‐ GGGGCAAACAGTTCTGTCTC‐3’] and reverse primer [5’‐AGAGCCACCATTTGTACCCA‐3’]. PCR products were checked on agarose gels and sequenced on an ABI 3,730 DNA sequencer (Applied Biosystems, Foster City, CA, USA). The sequencing results were compared with gene reference sequences in the UCSC hg19 to confirm potential mutations. NM_000117.2 was used as the reference sequence for the coding regions of the *EMD* gene.

## 3 RESULTS

### 3.1 Clinical findings

**Figure 1:**
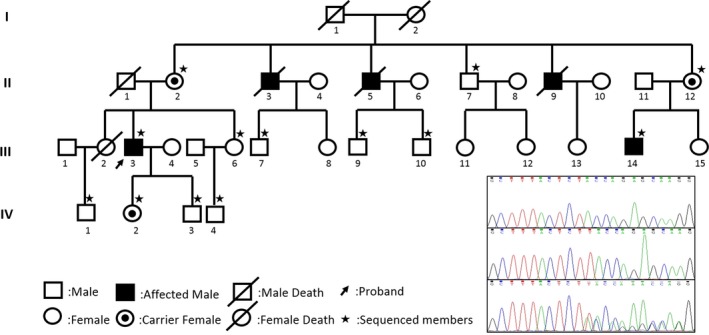
Pedigree of the family and mutation in EMD gene. Sanger sequence revealed a 1‐base insertion at c.253 in exon 3 of EMD

**Figure 2:**
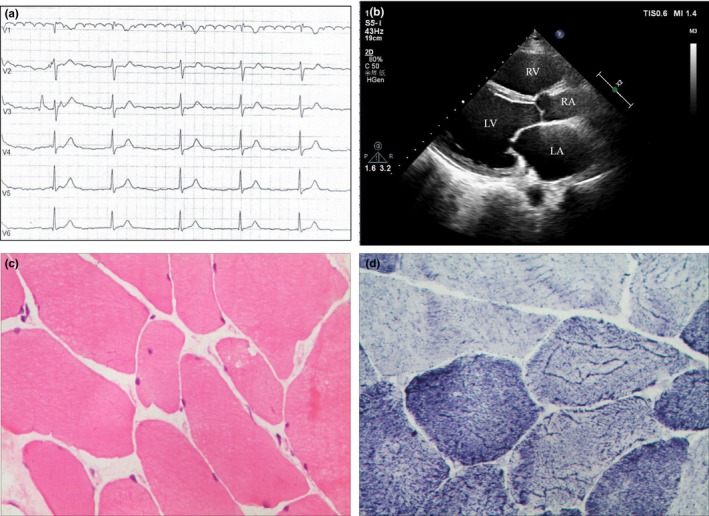
Clinical data of the proband. (a) Electrocardiogram showed atrial fibrillation with second degree atrioventricular block. (b) Chest echocardiography revealed generalized cardiomegaly. (c) Muscle histopathology (×400). HE staining showed mild fiber atrophy with irregular variation in size. (d) Muscle histopathology (×400). The mitochondria of type I and II muscle fiber presented fascicular structures with peripheral particles accumulated

The proband (III.3 in Figure 1), a 50‐year‐old man, who suffered from occasional palpitations, lower extremities edema and anhelation for over 10 years. In the year 2007, he was admitted to the cardiology department at the first time for auriculo‐ventricular block (Figure 2a). Echocardiography (Figure 2b) revealed generalized cardiomegaly. Serum creatine kinase (CK) level was normal (232.7 U/L, reference range 50–310 U/L). Physical examination showed no significant muscular weakness in his extremities, no scoliosis or contracture of any joints. An initial diagnosis of conduction cardiomyopathy was made. Inquiry of his complementary family history revealed that three of his uncles had sudden death during ages of 48–58 years old and one of his cousins had chest discomfort at 35 years old.

### 3.2 Genetic findings

Mutation analysis identified an 1‐bp insertion (c.253_254insT) in *EMD* gene exon 3, the frameshift hemizygous mutation resulting in a premature stop codon (p.Y85Lfs*8). Familial aggregation analyses revealed the mutation presented in 5 family members, including two hemizygotes (III.3, III.14) and three female carriers (II.2, II.12, IV.2). The propagating analysis revealed the mother carried the *EMD* heterozygous mutation. Subsequently, the patient's symptomatic cousin took examinations and found elevated CK level (458.9 U/L, reference range 50–310 U/L) and roughly normal electrocardiography (ECG). All the female carriers and his unaffected son had normal ECG and CK levels. The results verified the genotypes co‐segregated with phenotypes.

Half a year later, the proband received muscle biopsy on the left deltoideus triangularis. Pathological diagnosis showed myogenic damage, which was conformed to myogenic fibrous myopathy (Figure 2c and d). The follow‐up of the proband showed inconspicuous muscle weakness or joint contractures until now.

## 4 DISCUSSION

X‐linked EDMD is mainly caused by *EMD* mutations. The *EMD* gene (NM_000117.2) is mapped to chromosome Xq28 and contains 6 exons. The gene *EMD* encodes a ubiquitous nuclear membrane protein emerin consisting of 254 amino acids, which participates in membrane anchorage to the cytoskeleton (Bione et al., 1994). It was reported that cardiac involvement is the most important clinical symptom among patients with *EMD* mutations (Ura et al., 2007; Yuan et al., 2014). Cardiomyopathy appears in early adulthood and usually manifests as heart conduction block. Cardiac involvement usually arises after the second decade of life (Ben Yaou et al., 2007). ECG abnormalities occurrence often precede muscle disease. Cardiac conduction block can occur as early as 12 years of age and its severity is not related to the amount of muscular weakness and contractures (Sumitani, Ishikawa, Ishikawa, & Minami, 1995). Lack of emerin levels in heart may alter electrophysiology and myocardial cell adhesion, which could lead to conduction block (Sakata et al., 2005).

X‐linked EDMD associated with *EMD* mutations is clinically heterogeneous, ranging from mild to severe forms (Meinke et al., 2015). Different from the typical clinical features, the proband in our study had a medical history of severe arrhythmia and dilated myopathy, while inconspicuous skeletal muscular weakness and normal serum creatine kinase level. The other affected male (III.14), who presented paroxysmal cardiopalmus and slight limb weakness, had currently normal ECG, should be under close monitoring of cardiac function. Prognosis in EDMD is strongly associated with the severity of cardiac involvement. Continuous follow‐up of cardiac function is essential, including female carriers even in the absence of distinct clinical signs. It is crucial to provide prompt cardiac intervention and avoid unexpected lethal heart diseases for these particular patients. This case reminds us of the possibility of EDMD, while encountering young patients with significant cardiac conduction defects of unknown etiology, even if they have neither obvious skeletal muscle disorder nor joint involvement.

There are only few case reports on the predominant cardiac features of EDMD patients with *EMD* mutations (Karst, Herron, & Olson, 2008; Sakata et al., 2005; Vohanka et al., 2001; Yuan et al., 2014; Zhang et al., 2014) (Table 1). Learning from the previous reports, we knew that *EMD* mutations might be responsible for independent cardiac phenotype with severe heart rhythm defects or presenting as dilated cardiomyopathy, while muscle involvement and joint contractures were mild, localized, or seldom noticeable until the mutation been identified. It is interesting to find that four of the six reported patients were from Asia, whether the atypical X‐linked EDMD manifestations were due to incomplete penetrance or racial differences are still unknown. Our findings increase the case numbers of these rare clinical features and may provide research value of Asian patients in EDMD. Although *EMD* gene is specifically associated with X‐linked EDMD, the genotype–phenotype correlations, with special reference to cardiac involvements, still need to be further investigated.

**Table 1:** *EMD* mutations identified in studies with predominant cardiac phenotype

Proband	Gender/Age	Onset age	Electrocardiogram	Muscle involvement	Joint contracture	Nucleotide change	Amino acid alteration	Country	References
1	M/22	19	AVB	Neck(+)	neck (+)	c.450−2A>G	–	Japan	Yuan et al. (2014)
2	M/14	14	AVB; SA	(±)	Posterior neck(+)	c.667G>A	p.W226*	Japan	Sakata et al. (2005)
3	M/20	18	AVB；SB	(±)	Posterior neck(+)	c.667G>A	p.W226*	Japan	Sakata et al. (2005)
4	M/31	16	AVB; JB	Humeroperoneal(+)	(–)	c.712_716delGTCCT	p.V238Lfs*10	Czech Republic	Vohanka et al. (2001)
5	M/41	17	SB	Arms(+)	spinal column (+)	c.26_39delATACCGAGCTGACC	p.T10Lfs*18	China	Zhang et al. (2014)
6	M/57	33	SB；AF	(–)	(–)	c.106_108delAAG	p.K36–	USA	Karst et al. (2008)
7	M/50	40	AVB	(+)	(–)	c.253_254insT	p.Y85Lfs*8	China	Xu et al.

M: male; AVB: atrioventricular block; SA: Sinus arrest; JB: junctional bradycardia; SB: sinus bradycardia; AF: atrial fibrillation; (+): presence; (–): absence; (±): suspicious presence.

In conclusion, we describe a novel mutation of *EMD* gene, which provides genetic diagnosis for the atypical X‐linked EDMD individuals of the family. We provided scientific guidance and promote early intervention for EDMD patients, which may minimize the harmful effects of the disease.

## CONFLICT OF INTEREST

None Declared.
